# Instrumented treadmills: establishing measurement properties is necessary for evaluating clinical interventions

**DOI:** 10.1186/1757-1146-6-S1-P12

**Published:** 2013-05-31

**Authors:** Lloyd Reed, Stephen Urry, Scott Wearing

**Affiliations:** 1 School of Clinical Sciences, Queensland University of Technology, Kelvin Grove, Qld, 4059, Australia; 2 Faculty of Health Sciences and Medicine, Bond University, Gold Coast, Qld, 4229, Australia; 3 Centre of Excellence for Applied Sports Science Research, Queensland Academy of Sport, Qld, 4109, Australia

## Background

Instrumented treadmills that provide basic gait parameters in near real–time are emerging as valuable outcome tools in both clinical and research settings. Significant changes in step length and peak vertical force in the order of 2cm and 20-70N have been reported with footwear interventions and neurological disorders using these systems. However, published data about the systems’ measurement properties is lacking. This study evaluated the within– and between day repeatability of spatiotemporal parameters and vertical ground reaction forces (vGRF) measured by a new instrumented treadmill system.

## Methods

Thirty three healthy adults (mean age, 21.5±2.8 years) walked on an instrumented treadmill system (FDM–THM–S, Zebris Medical GmbH) at preferred speeds, on three separate occasions. Spatial, temporal and vGRF were collected over a 30–second capture period. Repeated measures ANOVAs were used to assess between–session differences in gait parameters, while agreement within- and between days were evaluated using 95% limits of agreement.

## Results

Statistically significant differences were found for the majority (14/16) of temporospatial and kinetic gait parameters over the three sessions (*P*<.01). The minimum change that could be detected with 95% confidence ranged from 3-16% for temporal parameters, 12-32% for spatial parameters, and 5-18% for kinetic parameters.

## Conclusion

Changes in gait parameters measured by the same treadmill and previously attributed to clinical interventions and neuromuscular pathology, fall within the measurement error of the treadmill determined in this study. The findings highlight the importance of determining the measurement properties of outcome tools in evaluating clinical interventions.

